# Extensive Fusion of Mitochondria in Spinal Cord Motor Neurons

**DOI:** 10.1371/journal.pone.0038435

**Published:** 2012-06-06

**Authors:** Geoffrey C. Owens, Elisabeth C. Walcott

**Affiliations:** The Neurosciences Institute, San Diego, California, United States of America; Boston University, United States of America

## Abstract

The relative roles played by trafficking, fission and fusion in the dynamics of mitochondria in neurons have not been fully elucidated. In the present study, a slow widespread redistribution of mitochondria within cultured spinal cord motor neurons was observed as a result of extensive organelle fusion. Mitochondria were labeled with a photoconvertible fluorescent protein (mitoKaede) that is red-shifted following brief irradiation with blue light. The behavior of these selectively labeled mitochondria was followed by live fluorescence imaging. Marking mitochondria within the cell soma revealed a complete mixing, within 18 hours, of these organelles with mitochondria coming from the surrounding neurites. Fusion of juxtaposed mitochondria was directly observed in neuritic processes at least 200 microns from the cell body. Within 24 hours, photoconverted mitoKaede was dispersed to all of the mitochondria in the portion of neurite under observation. When time lapse imaging over minutes was combined with long-term observation of marked mitochondria, moving organelles that traversed the field of view did not initially contain photoconverted protein, but after several hours organelles in motion contained both fluorescent proteins, coincident with widespread fusion of all of the mitochondria within the length of neurite under observation. These observations suggest that there is a widespread exchange of mitochondrial components throughout a neuron as a result of organelle fusion.

## Introduction

Human brain activity consumes about the same amount of adenosine trisphosphate (ATP) as leg muscles engaged in running a marathon [1]. Most of the ATP used by neurons is required for the maintenance of membrane excitability and to power synaptic transmission, and comes primarily from oxidative phosphorylation taking place in mitochondria [2]. Mitochondria are distributed throughout the axon and dendrites of neurons, and may be located from many hundreds of micrometers to as far as a meter away from the cell soma. The high demand for ATP and calcium buffering in neurons may lead to an increase in free radical formation in synaptic mitochondria rendering them more vulnerable to oxidative damage [3]. The challenge for neurons therefore is to maintain healthy mitochondria at considerable distances from the cell body.

It has been estimated that components of brain mitochondria have a half life of 26.3–31.6 days [4], [5]; thus the turnover of mitochondria in neurons may be quite slow. Since the mammalian mitochondrial genome encodes less than one percent of the organelle proteome [6], [7], synthesis of nearly all new mitochondrial proteins must occur outside the organelle, either locally at a synapse or node, or in the cell body. If synthesis of new mitochondrial proteins is primarily restricted to the cell soma, then exchange of mitochondria between processes and the soma must occur to replace damaged organelles. Rapid anterograde and retrograde transport of mitochondria in axons and dendrites of cultured neurons has been well documented [8], [9], [10], suggesting that this may be the mechanism of mitochondrial replenishment. In one study, more depolarized mitochondria (i.e. presumptively defective organelles) were observed to move towards the cell body [11].

On the other hand, the majority of mitochondria in axons and dendrites appear to be relatively stationary [12], [13] held in check by specific protein: protein interactions [14], [15], [16]. This implies that local protein synthesis would be required to restore such immobilized organelles, which is supported by evidence for local translation in dendrites [17], and the presence of ribosomes and mRNAs in axons [18]. Furthermore, DNA synthesis in axonal mitochondria has been documented [19].

A third possibility is that mitochondria traveling from the cell body fuse with immobilized dendritic or axonal mitochondria providing new components, including copies of the organelle genome. In non-neuronal cells this clearly occurs; mitochondria form a dynamic network that is constantly being reshaped by fusion and fission events [20], [21]. A fission event can result in one daughter mitochondrion with a low membrane potential and one with a high membrane potential, indicating that the damaged contents of the parent mitochondria are segregated in order to preserve function of one of the daughter organelles [22].

The critical importance of mitochondrial fusion and fission in the nervous system has been firmly established [23], [24]. In the human population, mutations in *OPA1* and *MFN2*, which are required for fusion, cause degeneration of the optic nerve and motor neurons respectively [25], [26]. The selective effect on long range projection neurons in these diseases underscores the significance of fusion to the maintenance of healthy mitochondria at distant nodes and synapses.

To investigate the dynamics of mitochondria in spinal cord motor neurons, a photoconvertible fluorescent protein [27] was used to label a subpopulation of organelles within cell bodies and neuritic processes. The fate of marked mitochondria was then followed by fluorescence microscopy. Short-term time lapse imaging captured rapidly moving mitochondria, while long-term imaging revealed extensive fusion of mitochondria in neurites and cell bodies, which resulted in a widespread dispersion of the contents of the marked mitochondria.

## Results

### Fusion of Mitochondria in Neuronal Cell Bodies

**Figure 1:**
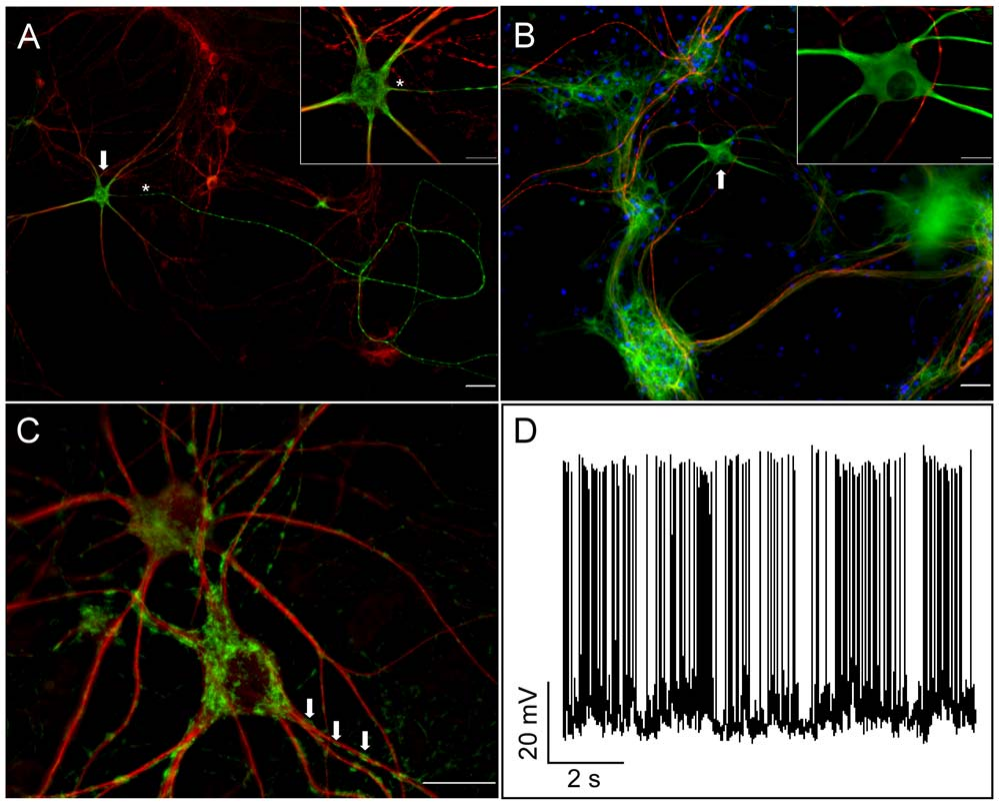
Characterization of motor neurons in spinal cord cultures. Antibodies were used to confirm the identity of the large neurons growing in the spinal cord cultures (24–29 div). A) A motor neuron (arrow) identified by staining for the non-phosphorylated neurofilament epitope recognized by SMI-32 mAb (green), and a polyclonal antibody against MAP2 (red). The presumptive axon is marked by an asterisk. B) A motor neuron (arrow) visualized with an anti-beta tubulin III antibody (green) does not express the phosphorylated neurofilament epitope recognized by SMI-312 mAb (red), a marker used to identify axons. Nuclei are labeled with DAPI. Scale bars correspond to 50 microns and 20 microns (higher power insets) respectively. C) An infected motor neuron immunostained with SMI-32 mAb (red) showing mitochondria expressing mitoKaede fluorescent protein (green). Arrows point to a close association of mitochondria with neurofilaments. Scale bar corresponds to 20 microns. D) A representative recording of spontaneous activity from an infected spinal cord motor neuron.

Several fluorescent proteins have been developed whose emission maxima are red-shifted following a brief irradiation with blue light [28]. In the present study, a mitochondrial targeting sequence from subunit VIII of cytochrome c oxidase was added to the Kaede protein [27] in order to mark subpopulations of mitochondria in cultured spinal cord motor neurons. Motor neurons were identified by size and morphology, and this was later confirmed by immunocytochemistry (Figure 1A–C). Figure 1D shows a trace of action potentials generated by an infected motor neuron measured by whole cell patch clamp, confirming that infected cells are spontaneously active under the culture conditions used.

To selectively mark mitochondria within the somas of individual motor neurons, the microscope aperture was adjusted so that the light path was restricted to an opening approximately 30 microns in diameter. The opening was aligned over a motor neuron cell body, and the culture was briefly illuminated with blue light (>405 nm) to convert the exposed mitochondria from fluorescing green to fluorescing red. Using a 300W DG-4 Xenon light source, an exposure time of one minute was sufficient to photo-convert most of the mitoKaede protein. Illumination times over two minutes were deleterious; several hours post-illumination all of the photoconverted mitochondria appeared spherical in shape rather than elongated, and had not fused with green fluorescing mitochondria. Variables that affected the extent of photoconversion included the thickness of the cell and the level of expression of the fluorescent protein. This latter variable also affected cell viability; neurons expressing high levels of fluorescent protein were more sensitive to phototoxicity.

In one experiment neurons were infected with the mitoKaede virus and a second lentivirus encoding a short-lived green fluorescent protein (GFP) to mark the cell soma. Images were taken every 10 seconds for 30 minutes within a minute of exposing a single motor neuron to blue light. A portion of the time lapse series was rendered as a QuickTime movie at 10 frames per second, and as shown in Movie S1, movement of individual mitochondria into and out of the cell body was apparent. The filter sets used to acquire the red and green fluorescent images minimized any spillover of signal between channels (see Materials and Methods).

**Figure 2:**
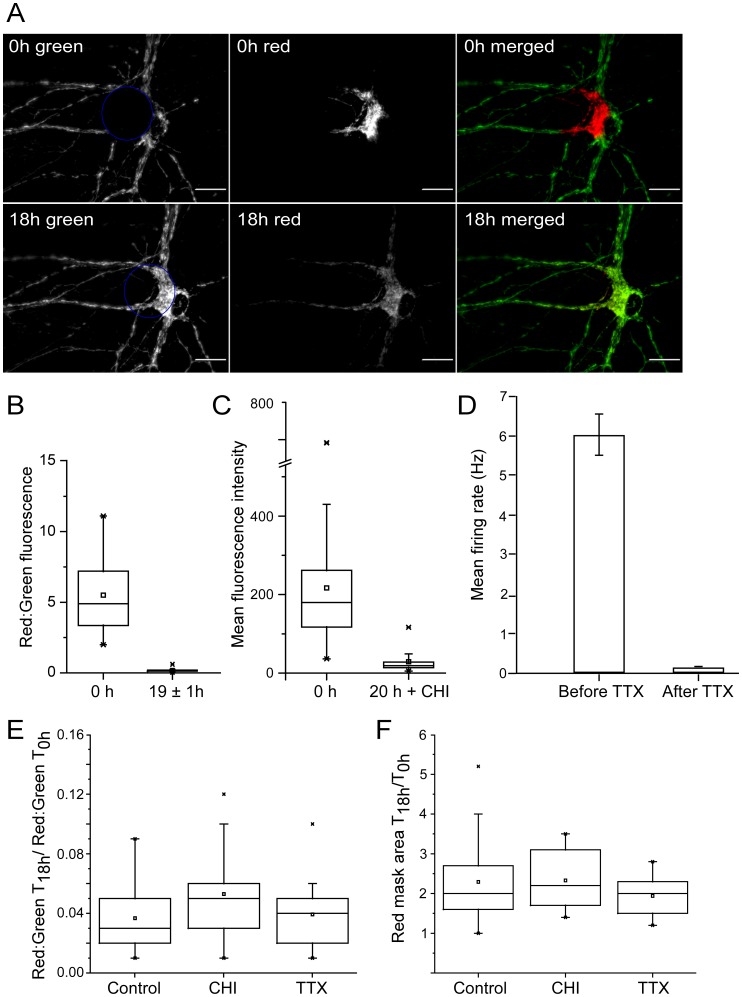
Constitutive redistribution of labeled mitochondria. A) Images of a living motor neuron (24 div) were taken within a minute after photo-converting the mitoKaede protein, and then after 18 hours. Over a period of 18 hours mitochondria from the periphery appeared to extensively fuse with organelles from the cell body. Blue circles in the leftmost panels delineate the area of the neuron that was exposed to blue light. Relative fluorescence intensity levels are directly comparable. Scale bars correspond to 20 microns. B) Quantitation of the redistribution of red and green fluorescent mitoKaede. The mean fluorescence intensity per pixel was calculated for both red and green pixels in masks generated solely based on the distribution of red pixels. For each cell (n = 33), the ratio of red: green mean pixel intensity at time 0 h and after 18 h (19±1 h) is plotted as a box and whisker plot. A decrease in this ratio reflects a dilution of the finite amount of red fluorescent mitoKaede with green fluorescent mitoKaede. The median ratio changed from 4.9 to 0.17. C) The level of expression of a short-lived GFP in motor neurons before and after treatment with CHI was quantified by manually generating masks that encompass the green pixels in the cell soma, and calculating the mean fluorescence intensity (n = 10; p<0.001). D) Patch clamp recordings were performed on infected spinal cord neurons sampled from 4 coverslips (n = 7). Spontaneous spiking activity was recorded over 20 s, and action potentials were blocked in all 7 cases by 1 µM TTX. Data are plotted as mean firing rate of the same cells before and after addition of TTX to the ACSF (p<0.001). E) Pooled data showing no significant effect of cycloheximide (CHI 10 µg per ml, n = 20) or tetrodotoxin (TTX, 1 µM, n = 16) on the redistribution of labeled mitochondria over time compared to untreated motor neurons. For each cell in each treatment group, the ratio of red: green mean pixel intensity after 18 h (19±1 h) is normalized to the starting ratio in the same cell, and plotted as a box and whisker plot. F) Box and whisker plots of the change in the size of masks generated from the distribution of red pixels reflecting the dispersion of red fluorescent mitoKaede over time.

To investigate longer term changes in the distribution of mitochondria, multiple cultures containing photoconverted mitochondria in the cell bodies of several motor neurons were visualized immediately after exposure to blue light and again approximately 18 hours later (19±1 h; n = 33). A Z-stack of images in increments of 0.2 microns, encompassing the entire volume of the cell, was taken at each time point using identical exposure times. As exemplified by the motor neuron shown in Figure 2A, the distribution of green and red fluorescing mitochondria changed over time. After 18 hours the focal area of red fluorescence in the cell body was no longer discernible, and had been replaced by a mixture of red and green fluorescent mitoKaede. Meanwhile the red fluorescence had spread from the cell soma into the neurites. Since the fluorescent protein was targeted to the matrix of the mitochondrion, it appeared that extensive fusion must have taken place between mitochondria from the cell soma and attached neurites in order to account for the observed redistribution of red and green fluorescing proteins. In Figure 2A, the red fluorescence intensity scale was adjusted to the same upper and lower values in both images, and likewise for the green fluorescence intensity scale so that relative expression levels are directly comparable.

To obtain an objective measure of the redistribution of fluorescent protein, the Ridler Calvard algorithm was used to generate masks calculated from the distribution of red pixels in projection images of each motor neuron. The average intensity of red and green pixels within these masks was computed together with the extent of overlap between them (Pearson correlation coefficient). In these imaging studies a pixel corresponded to 0.213 microns based on the numerical aperture and magnification of the lens. The ratio of the mean red to green pixel intensities provided a measure of the relative concentration of red mitoKaede to green mitoKaede within mitochondria. This ratio significantly decreased (p<0.001) after 18 hours reflecting a mixing of green mitoKaede with the finite amount of red mitoKaede (Figure 2B). At the same time the size of the mask encompassing the red pixels increased, reflecting the spread of the red mitoKaede (Figure 2F). This increase may be an underestimate because some red pixels in mitochondria farthest away from the cell body may have fallen below the threshold used to calculate the mask. The Pearson correlation coefficient between red and green pixels within the mask increased from 0.17±0.19 to 0.83±0.15 suggesting that many more red and green mitoKaede fluorescent protein molecules were within a micron of each other, increasing the likelihood that they were in the same organelle.

To rule out the possibility that *de novo* synthesis of mitoKaede could account for the observed increase in green fluorescence in the cell body, cycloheximide (CHI, 10 µg per ml final concentration) was added. To confirm the efficacy of the drug treatment, a separate culture was infected with a virus that encodes the short-lived GFP, referred to previously. The GFP fluorescence intensity in 10 cells was measured before and after addition of cycloheximide. As shown in Figure 2C, inhibiting protein synthesis significantly (p<0.001) reduced the level of expression of this short-lived GFP. Data from two independent experiments showed that inhibiting protein synthesis did not significantly alter the redistribution of red and green mitoKaede (n = 20; Figure 2E, p = 0.176, and Figure 2F, p = 0.762). The Pearson correlation coefficient increased from 0.192±0.234 to 0.814±0.0945.

The redistribution of mitoKaede was also unaffected by blocking neural activity with 1 µM tetrodotoxin (TTX). Whole-cell patch clamp recordings made from infected neurons before and after addition of TTX confirmed the efficacy of the treatment (Figure 2D; n = 7, p<0.001). Data from two independent experiments showed that inhibiting action potentials did not significantly alter the redistribution of red and green fluorescent proteins in motor neurons (Figure 2E; n = 16, p = 0.615, and Figure 2F, p = 0.216). The Pearson correlation coefficient increased from 0.0413±0.231 to 0.854±0.155. This would suggest that the extensive organelle fusion implied by the redistribution of mitoKaede is a constitutive process.

### Bi-directional Fusion of Mitochondria within Processes

**Figure 3:**
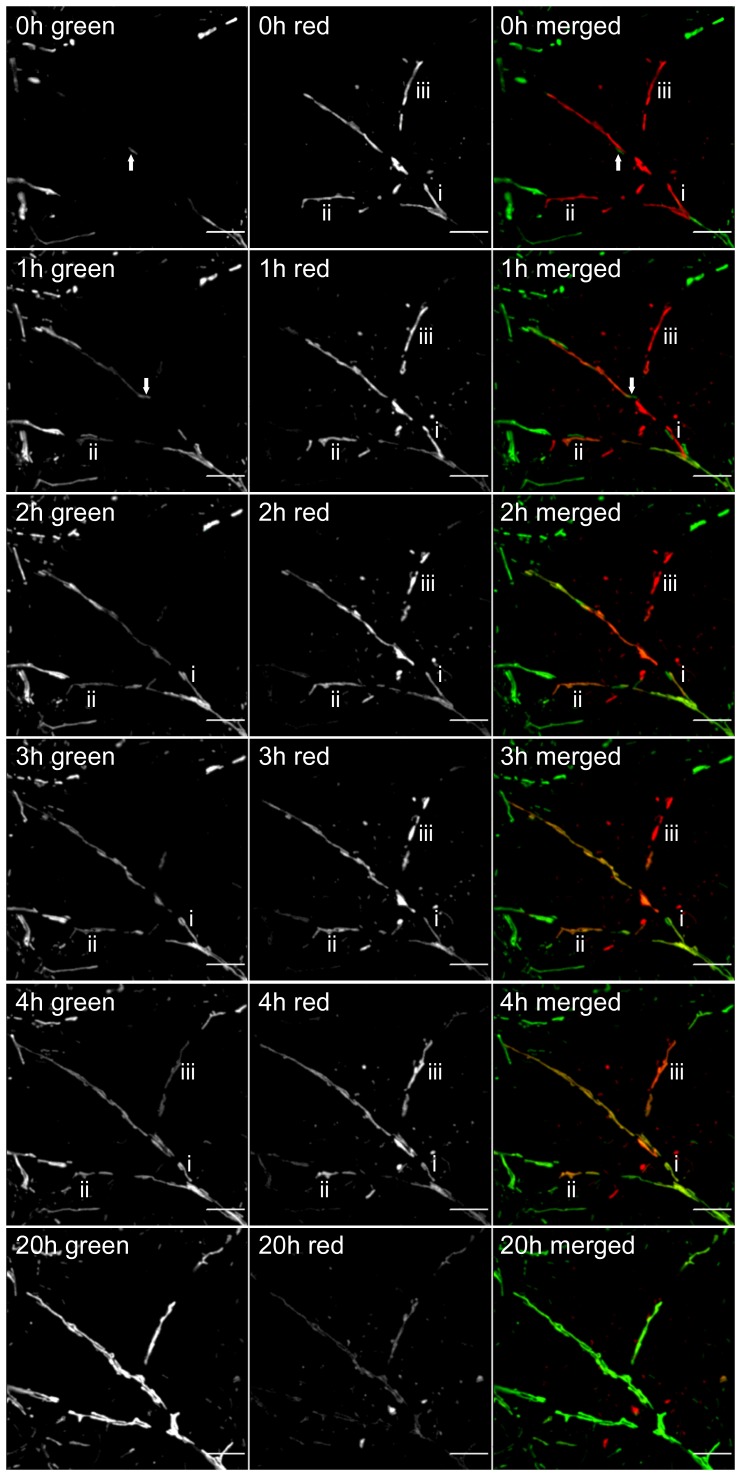
Time course of the fusion of individual mitochondria. A living neurite (28 div) with mitochondria containing photoconverted mitoKaede was imaged over several hours. The appearance of green fluorescing mitoKaede within individual red fluorescing mitochondria (labeled i- iii) during 4 hours of observation is seen. The arrow indicates the location of a mitochondrion that had entered the field of view before the first image was taken. By 20 hours, the red fluorescent protein was distributed throughout the mitochondria under observation. Scale bars correspond to 5 microns.

The reproducible change in the pattern of red and green mitoKaede expression strongly implied that widespread fusion between organelles from the soma and neurites takes place leading to a mixing of their contents over time. The limits of far field fluorescence microscopy employed in this study precluded definitively resolving individual mitochondria that had undergone fusion within the cell body of a motor neuron. On the other hand it was possible to follow the fusion of individual organelles within surrounding neurites. A circle ∼30 microns in diameter, and at least 200 microns from an identified cell body, was irradiated with blue light to mark mitochondria in a single neuritic process. Spinal cord motor neurons elaborate multiple branched dendrites and a single long axon, which may form collaterals [29], [30], and as shown in Figure 1 the cultured motor neurons used in this study were highly polarized. However, in living cultures expressing mitoKaede it was not possible to unequivocally distinguish the axon from long dendrites, but given the greater prevalence of the latter, it was more likely that a given neurite under observation was a dendrite. Fluorescence images were taken every hour for four hours and then again after 20 hours. As exemplified in Figure 3 there was clear evidence of fusion between juxtaposed mitochondria over time. The fate of individual mitochondria could be followed over the 4 hour period (mitochondria are labeled i-iii in Figure 3). Between the time of blue light irradiation and image acquisition, a single green mitochondrion had moved into the territory occupied by the red mitochondria but had not fused (arrow). The amount of red fluorescent protein in mitochondria in the photoconverted area was reduced after 20 hours, whereas all of the organelles in the field of view contained much more green fluorescent protein (Figure 3). This implied that the contents of the original red fluorescing mitochondria had been dispersed by multiple fusion events with green fluorescing mitochondria coming from outside the region under observation. At the same time the photoconverted protein had spread to mitochondria away from the original site of photoconversion.

### Rapid Movement and Slow Fusion of Mitochondria Occur within the Same Neuritic Process

**Figure 4:**
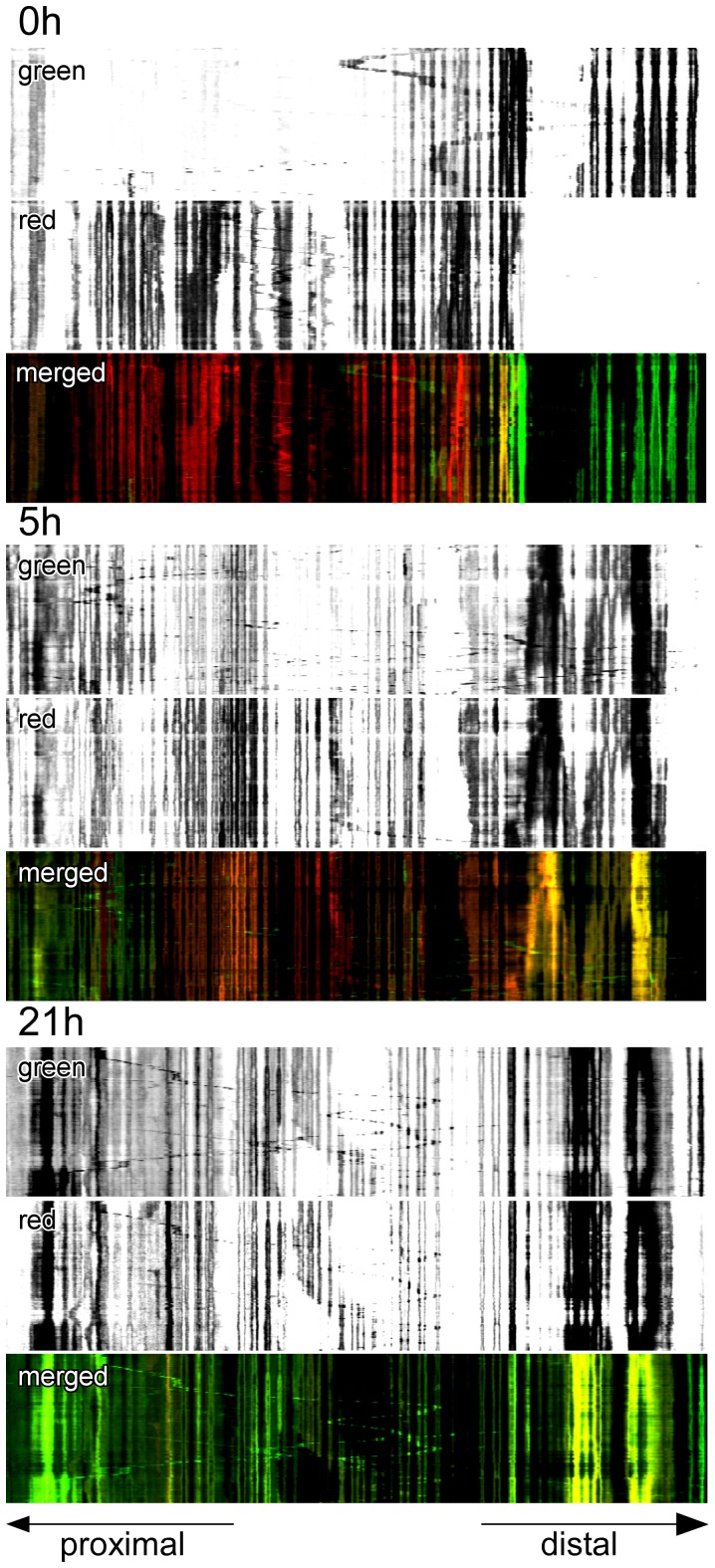
Slow fusion and rapid movement of mitochondria within the same neurite. Time lapse images were taken every 10 seconds for 30 minutes at 0 h, 5 h, and 21 h following the marking of a subpopulation of mitochondria within a living neurite (56 div). The data are presented as kymographs, and show a progressive fusion of all of the mitochondria under observation with organelles originating from the proximal (cell body) and distal sides of the process. At each time point a limited number of mitochondria move during the time lapse imaging. After 21 hours, moving organelles appear to be the product of fusion events.

**Figure 5:**
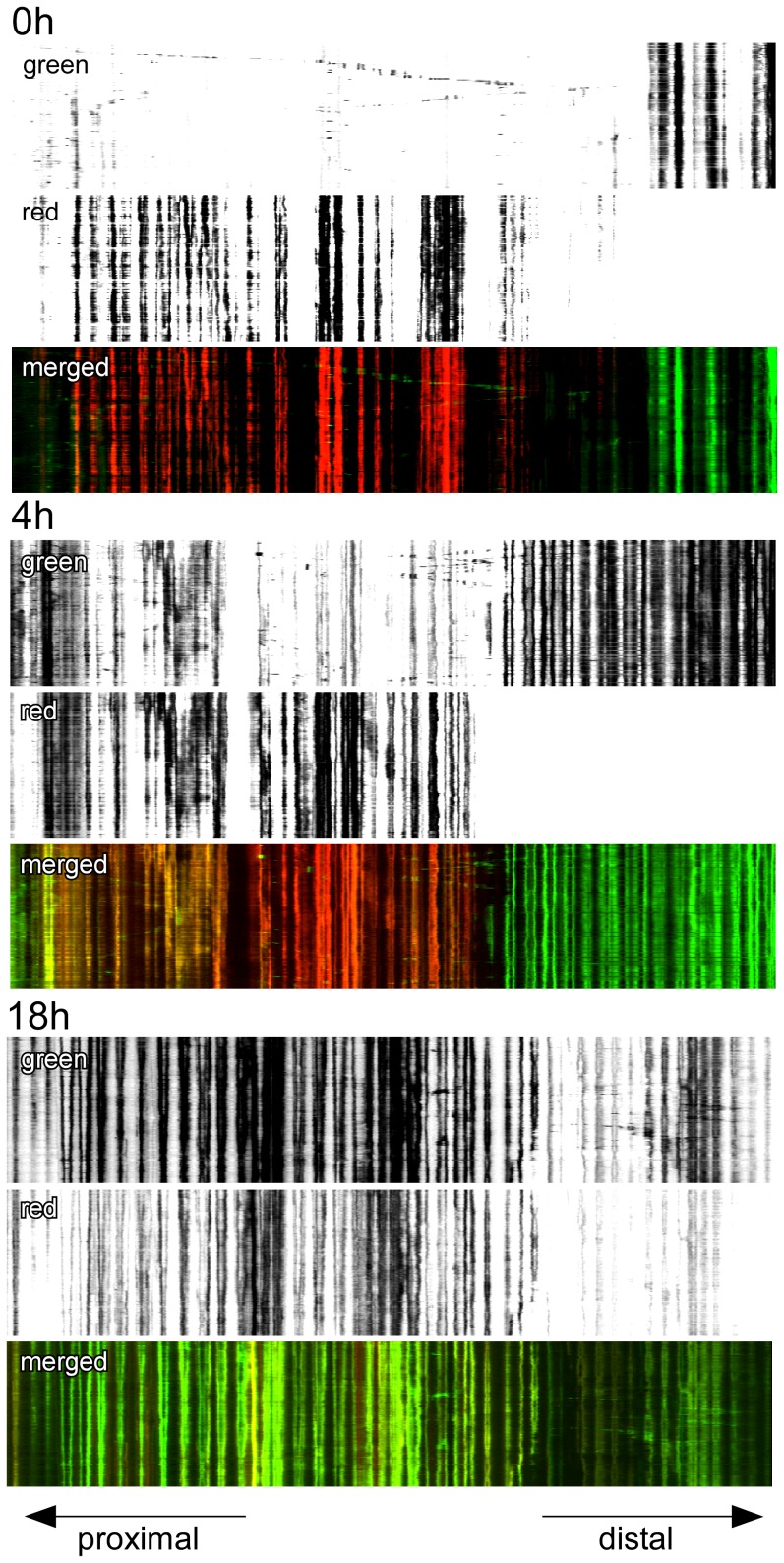
Inhibition of neuronal activity with TTX does not affect mitochondrial fusion in neurites. Kymographs show that fusion of mitochondria was unaffected by continuously blocking neuronal activity with TTX (1 µM final concentration). Time lapse images were taken every 10 seconds for 30 minutes at 0 h, 4 h, and 18 h following the marking of a subpopulation of mitochondria within a living neurite (27 div).

In the course of imaging individual neurites, fusion events were captured in time lapse images taken in the first 30 minutes following exposure to blue light. Portions of two time lapse series are shown in Movies S2 and S3. In Movie S2, a slow moving mitochondrion is seen to fuse with a second mitochondrion approximately 10 microns away; in Movie S3, a highly motile organelle enters the field of view and fuses with a stationary organelle. To visualize both rapid movement and fusion over time, images of photoconverted mitochondria in a length of neurite were acquired every 10 seconds for 30 minutes and then again after 5 hours, and 21 hours. The time lapse images were plotted as kymographs (Figure 4) and were also converted into QuickTime movies (Movie S4, Movie S5, and Movie S6). By 5 hours, fusion of red fluorescing mitochondria with green fluorescing mitochondria entering from both the proximal and distal directions was clearly evident, and after 21 hours the entire field of view was occupied by mitochondria containing both red and green fluorescent protein. Superimposed upon this process, moving mitochondria were apparent at all time points traversing the approximately 140 microns of neurite under observation. In the first 5 hours following irradiation motile red and green fluorescing mitochondria were discernible; by 21 hours moving mitochondria co-expressed both red and green fluorescent protein, indicating that they were the product of a fusion event that had occurred in the intervening time. Adding TTX did not appear to alter the fusion of mitochondria within neurites (Figure 5). Particle tracking was used to determine the velocity of moving mitochondria, including the mitochondria that were observed to fuse (Movies S2 and S3). Measurements were made of individual mitochondria that moved without stopping in consecutive images taken from 12 independent time lapse series. The extent of continuous movement, either towards or away from the cell body, varied from only 5 microns to 58.9 microns and the average speed was 0.19±0.1 microns per second (n = 14). Maximum velocities ranged from 0.16 to 0.79 microns per second.

## Discussion

A number of studies have explored the dynamics of mitochondria in living neurons [11], [12], [31], [32], [33], [34]. In the present study, photoconvertible mitoKaede was used to show that widespread fusion of mitochondria occurs in living motor neurons over several hours. It was originally envisioned that this approach could be used to follow the fate of individual mitochondria moving in an anterograde direction over long distances. In Movie S1, trafficking of photoconverted mitochondria out of the cell soma is clearly seen. However, after approximately 18 hours it appeared that essentially all mitochondria in the cell body had fused with organelles entering from the surrounding neurites. Treatment of cultures with CHI ruled out *de novo* synthesis as an explanation for the increase in green fluorescing protein seen over time in mitochondria found in the cell body. Blocking spontaneous activity with TTX did not appear to alter the extent of mitochondrial fusion suggesting that the process is not directly linked to activity-dependent energy demand. On the other hand, it was recently found that the rate of mitochondrial fusion in motor neurons is decreased by over-expression of a disease-causing SOD1 mutant, suggesting that the process is susceptible to pathological changes in these neurons [34].

In neurites, it was evident that fusion between closely apposed mitochondria led to a local mixing of red and green fluorescent proteins (Figure 3). The dilution over time of a finite amount of red fluorescent protein in a small number of photoconverted mitochondria suggested that multiple fusion events occurred as the contents of these mitochondria were distributed among a greater number of organelles. Although the motor neurons were highly polarized into axons and dendrites (Figure 1), it was not possible to distinguish between these two types of neuritic process in living neurons. The pattern of fusion that we observed may be confined to dendrites in which mitochondria are known to form an extensive network [35]. Note that recent work indicates that mitochondria within axons also fuse [34].

Although it has been shown that mitochondria move less in dendrites than in axons [12], the reported rates of mitochondrial transport in dendrites are comparable to those in axons [10]. In the time lapse experiments that were carried out motile green fluorescing mitochondria were observed to traverse the length of neurite occupied by red fluorescing mitochondria. Consonant with previous reports [10], the average speed of moving mitochondria was 0.19±0.1 microns per second. When the time lapse imaging was repeated after several hours, organelles in motion contained both fluorescent proteins, coincident with widespread fusion of mitochondria resident within the length of neurite under observation.

Under the culture conditions used here, neuritic mitochondria that do not appear to move during short-term time lapse imaging [13], [34] may be sufficiently motile to fuse with their immediate neighbors (Movie S2). Fusion between a highly motile mitochondrion and a stationary organelle was also observed (Movie S3) indicating that mitochondria do not exclusively fuse with their immediate neighbors. It remains to be investigated whether limited mitochondrial movement is dependent upon microtubules. In one study, it was reported that fusion of mitochondria did not depend upon an intact microtubule network [36].

Mitochondrial transport in neurons is normally dependent upon microtubules and involves interactions between the motor proteins, kinesin-1 and dynein, and adaptor proteins including the Milton-like proteins TRAK1 (OIP106) and TRAK2 (GRF-1), and Miro GTPases [9], [37], [38]. It has been shown that suppression of Miro1 in cultured hippocampal neurons and Miro2 (but not Miro1) in dorsal root ganglion neurons inhibits mitochondrial movement [15], [33]. Conversely, over-expression of Miro1 increases the number of moving mitochondria [15], and promotes a more thread-like morphology reflecting a change in the balance between fusion and fission [32]. Experiments using recombinant Miro with mutated EF hands have shown that both effects are negatively regulated by calcium [16], [32]. Miro has been shown to associate with Mitofusin 1 and 2, the mitochondrial proteins that mediate outer membrane fusion [33], supporting the idea that it may be involved in regulating fusion of mitochondria in neurons. It will be of interest to determine whether manipulating the levels of Miro proteins affects the widespread fusion of mitochondria in spinal cord motor neurons documented here.

It is also worth noting that mitochondria can associate with actin filaments via interaction with specific unconventional myosins [39], [40]. Time lapse imaging of cells over-expressing myosin XIX indicated that there may be enough movement of otherwise tethered mitochondria to allow fusion to occur between apposed organelles [39]. A role for neurofilaments also cannot be excluded [41].

In summary the present study has revealed that mitochondria have a propensity to fuse not only within the soma of cultured motor neurons but also in neuritic processes at a distance from the cell body. Although many mitochondria are transported rapidly, possibly to meet local energy demands, a slower constitutive process involving cycles of fusion, and potentially fission, also takes place. Thus mitochondria at distant synaptic and nodal sites may be the products of a chain of fusion and fission events that starts in the cell body. This widespread exchange of mitochondrial components could explain how a deleterious mutation arising in one mitochondrion’s genome could spread throughout a neuron’s organelle population leading to dysfunction or demise of the cell.

## Materials and Methods

### Ethics Statement

Non-survival animal surgeries were approved by The Neurosciences Research Foundation’s Institutional Animal Care and Use Committee, and were performed in strict accordance with the U.S. Public Health Service (PHS) Policy for Humane Care and Use of Laboratory Animals (PHS Animal Welfare Assurance no. A4558-01).

### Preparation and Infection of Spinal Cord Neurons

Spinal cord neurons were isolated from E14 day old rat embryos essentially as described [42] except that cells were plated onto glass bottomed culture dishes (MatTek Corporation) or on 12 mm circular glass coverslips (Carolina Biological) coated with poly-D-lysine/laminin (Roche Applied Science) in serum-free glia conditioned DMEM [13]. Cytosine arabinoside (Sigma Aldrich) was added after 12 days in vitro (div), and on day 14, the medium was exchanged for lipid-rich BSA-containing unconditioned low glucose serum-free DMEM supplemented with B27 without antioxidants (Life Technologies). On day 14 or 15 cultures were infected with a recombinant FIV lentivirus that encodes a Kaede fluorescent protein targeted to the mitochondrion. Cultures were used for imaging experiments from 21 div up to 60 div.

The gene for Kaede protein was amplified by PCR from a commercially available plasmid (MBL) and was combined with the mitochondrial targeting sequence from subunit VIII of human cytochrome c oxidase found in pEYFP-Mito (Clontech). The mitoKaede gene was inserted into the FIV vector FLX1.8CMV to generate FLX1.8CMVmitoKaede (Addgene plasmid no. 28133) In order to obtain optimal expression in motor neurons, the CMV enhancer was replaced by an enhancer sequence from the rat cytochrome c gene [43] to make FLX1.8CytmitoKaede. Virus was produced by transient transfection of 293T cells as previously described [13], and concentrated by centrifugation through Vivaspin 20 polyethersulfone ultrafiltration units (Sartorius). Based upon the expression of mitoKaede in transduced B104 cells, measured by flow cytometry, sufficient virus was added to infect essentially all of the neurons in a culture.

### Image Acquisition and Analysis

A Leica DMI6000B inverted fluorescence microscope with an automated stage, and connected to a CCD camera and 300W Lambda DG-4 xenon light source was used to acquire images of fluorescently-labeled mitochondria in living neurons [13]. Cells grown in a glass-bottomed culture dish were housed for approximately 24 hours in a stage top micro-incubator that is coupled, via tubing and a pump, to a large tissue culture incubator, which maintains a humidified atmosphere of 10% CO_2_ and 90% air at approximately 37°C [44]. Microscope functions were controlled by the SlideBook™ v5.0 software package (Intelligent Imaging Innovations). Fluorescing motor neurons were identified at low magnification (200X) based upon size and morphology, and the coordinates of their locations within the culture were recorded. Under high magnification (630X) suitable candidates for imaging were selected, and the aperture through which light travels was minimized and aligned over a cell body or a neurite. This allowed for a focal conversion of a subpopulation of mitochondria from fluorescing green to fluorescing red by exposure to light passing through a 405/10 nm single band pass filter for one minute. This exposure time was empirically determined, and was 50 percent of the illumination time that caused phototoxicity in some motor neurons. For each cell body that was imaged, a stack of images at 0.2 micron intervals was taken; for individual neuritic processes, single images were captured. For a time lapse series, images were acquired every 10 seconds for 30 minutes. Image acquisition times were kept as short as possible (100–250 ms for the 300W DG-4 xenon light source) to avoid photodamage resulting from repeatedly imaging the same cell. Green fluorescent mitoKaede was excited by light passing through a 485±12 nm single band pass filter, and photoconverted red fluorescent mitoKaede was excited through a 560±15 nm single band pass filter. Green fluorescence was captured through a matching 410/504/582/669 quad band dichroic mirror and a 525±18 nm single band pass filter. Red fluorescence was detected through the same quad band dichroic mirror and a 560±15 nm single band pass filter. All filter sets were from Chroma Technology.

All of the image processing and analysis was carried out using the tools in SlideBook™ v5.0. To generate images for display, nearest neighbor deconvolution was performed on a serial z-stack of images of a cell; a projection image using maximum pixel values was then generated. No neighbors deconvolution was used for single images of a neurite. The same scale of fluorescence intensity was used for each image so that expression levels could be directly compared visually. Images were exported as. tiff files, and figures were prepared in Canvas™ v12 (ACD Systems). Quantification was performed on the stacks of raw images after deconvolution. A mask encompassing pixels in the red channel was generated using the Ridler-Calvard algorithm and objects <50 pixels in size were excluded. The mean pixel intensities in the red and green channels were calculated as well as the Pearson correlation coefficient, which measured the extent of overlap of red and green pixels within the mask. Data were plotted and resulting graphs were exported to Canvas™ v12. Statistical calculations, Mann-Whitney non-parametric U test, and Students t-test were made using SofaStats (www.sofastatistics.com). For a particular time series, kymographs generated using the smooth curve analysis function in SlideBook™, were normalized to the same scale of fluorescence intensity, and exported as tiff files. Portions of 30 minute time lapse series were converted into QuickTime movies at 10 frames per second. The velocities of moving mitochondria were determined using the manual particle tracking tool in the SlideBook™ v5.0 software package. The position of the same mitochondrion in a sequence of consecutive time lapse images was tagged, and average velocity and maximum velocity were automatically calculated. Data were obtained from 14 mitochondria in 12 independent time lapse series.

### Patch Clamp Recordings

To record spontaneous activity from motor neuron cultures (26–27 div), coverslips were transferred from culture media into a submerged recording chamber mounted on a fixed-stage upright microscope (Leica DMLFSA), and were perfused with warmed (31°C) artificial cerebrospinal fluid (ACSF) at a rate of 3 ml/min, and allowed to equilibrate for 5 min before recording. The ACSF contained (in mM) 124 NaCl, 3 KCl, 1.25 NaH_2_PO_4_, 26 NaHCO_3_, 2 MgCl_2_, 2 CaCl_2_, and 10 dextrose. The osmolality was adjusted with sucrose, and the pH was buffered to 7.4 by bubbling 95%O_2_/5%CO_2_. Motor neurons were visually identified at 400X magnification using infrared differential interference contrast optics and an infrared-sensitive camera. Neurons were selected for recording according to published criteria [45], [46], which included having a soma that was >20 µm in diameter and two or more thick, branching processes. Whole-cell patch clamp recordings were made according to standard procedures, and as described [47], [48]. Briefly, whole-cell patch clamp recordings were made using pulled glass electrodes with resistances of 5–6 MΩ and tip diameters of 1 µm. The internal solution for the electrodes contained (in mM) 110 K-gluconate, 10 KCl, 10 (Na) phosphocreatine, 10 HEPES, 4 (Mg) ATP, 0.3 (Na) GTP, 5 EGTA. Osmolality was adjusted to 290 mOsm with sucrose, and pH was adjusted to 7.4 with KOH. Liquid junction potentials (5 mV) were left uncorrected. Neurons were included in the analysis if series resistances were <20 MΩ and input resistances were >100 MΩ. Signals were amplified using a Multiclamp 700A (Molecular Devices) then filtered at 4 kHz, digitized at 10 kHz, and acquired with a 16 bit digital signal processing board (dSpace Inc.) using custom software written in Matlab and Simulink (MathWorks) by N.S. Desai, The Neurosciences Institute.

### Immunocytochemistry

Cultures were fixed in 4% paraformaldehyde for 20 minutes at room temperature, and post-fixed with methanol (15 minutes at −20°C). Cells were blocked in 10% goat serum and incubated overnight at 4°C with monoclonal antibodies directed against non-phosphorylated neurofilament (SMI-32, 1∶5000; Covance), phosphorylated neurofilament (SMI-312, 1∶5000; Covance), beta tubulin III (1∶1000; Sigma), and polyclonal antibodies directed against MAP2 (1∶500; AbCAM). Alexa Fluor®, 488 and 568 secondary antibodies were added at room temperature for 2 hours (1∶200; Life Technologies).

## Supporting Information

Movie S1 **Movement of mitochondria into and out of the cell body of a spinal cord motor neuron.** Left panel, green fluorescence channel; middle panel, red fluorescence channel; right panel, merged.
(MOV)

Movie S2 **Fusion of two mitochondria in a motor neuron neuritic process.** Left panel, green fluorescence channel; middle panel, red fluorescence channel; right panel, merged.
(MOV)

Movie S3 **Fusion of mitochondria in a motor neuron neuritic process**. Left panel, red fluorescence channel; middle panel, green fluorescence channel; right panel, merged.
(MOV)

Movie S4 **Movement of mitochondria in a motor neuron neuritic process immediately after photoconversion.** Left panel, red fluorescence channel; middle panel, green fluorescence channel; right panel, merged.
(MOV)

Movie S5 **Movement of mitochondria 5 hours after photoconversion in the same length of neurite as in Movie S4.** Left panel, red fluorescence channel; middle panel, green fluorescence channel; right panel, merged.
(MOV)

Movie S6 **Movement of mitochondria 21 hours after photoconversion in the same length of neurite as in Movies S4 and S5.** Left panel, red fluorescence channel; middle panel, green fluorescence channel; right panel, merged.
(MOV)
